# Nutri-Epigenetics and Gut Microbiota: How Birth Care, Bonding and Breastfeeding Can Influence and Be Influenced?

**DOI:** 10.3390/ijms21145032

**Published:** 2020-07-16

**Authors:** Rosita Gabbianelli, Laura Bordoni, Sandra Morano, Jean Calleja-Agius, Joan G. Lalor

**Affiliations:** 1 Unit of Molecular Biology, School of Pharmacy, University of Camerino, 62032 Camerino, Italy; laura.bordoni@unicam.it; 2 School of Medicine and Midwifery, Department of Neurology, Ophthalmology, Maternal and Childhood Sciences, Genoa University, Largo R. Benzi, 10-16132 Genova, Italy; sandra.morano@unige.it; 3 Department of Anatomy, Faculty of Medicine and Surgery, University of Malta, MSD2080 Msida, Malta; jean.calleja-agius@um.edu.mt; 4 School of Nursing and Midwifery, Trinity College Dublin, 24 D’Olier Street, Dublin 2, Ireland

**Keywords:** nutri-epigenetics, gut microbiota, breastfeeding, birth care, best practice, parent education

## Abstract

Maternal lifestyle is an important factor in the programming of an infant’s epigenome, in particular when considered alongside the mode of birth and choice of feeding method (i.e., breastfeeding or formula feeding). Beginning in utero, and during the first two years of an infant’s life, cells acquire an epigenetic memory of the neonatal exposome which can be influential across the entire lifespan. Parental lifestyle (e.g., malnutrition, alcohol intake, smoke, stress, exposure to xenobiotics and/or drugs) can modify both the maternal and paternal epigenome, leading to epigenetic inheritance in their offspring. This review aims to outline the origin of early life modulation of the epigenome, and to share this fundamental concept with all the health care professionals involved in the development and provision of care during childbirth in order to inform future parents and clinicians of the importance of the this process and the key role it plays in the programming of a child’s health.

## 1. Introduction

Nutri-epigenetics is the study of the role of dietary components in the modulation of the epigenome [1,2]. Food oxidation produces different metabolites involved in the modulation of chromatin structure and in the regulation of gene expression. For example, acetyl groups from Acetyl-CoA are used by histone acetyltransferases (HATs) to neutralize the charges at chromatin level, by decreasing the electrostatic interaction between the positive charge of aminoacidic residues at the histone tails and the negative charge of phosphate groups of the DNA [3]. The acetylation of specific amino acidic residues of the histone tail is associated with open chromatin, thus it can turn on gene expression. Whereas deacetylation leads to condensation of chromatin and gene silencing. A range of effects on the modulation of gene expression can be derived from histone methylation, which takes place in the addition to one or more methyl groups at the aminoacidic residues of histone tails [1]. The enzymes involved in histone methylation are the histone methyltransferases (HMTs) which receive the methyl group from the *S*-adenosyl-methionine (SAM), the universal methyl donor, which is the final product of the one carbon cycle. In particular, the one carbon cycle combines the folic acid cycle with the methionine cycle to synthetize the SAM, which donates methyl groups to both DNA-methyltransferases (DNMTs) and HMTs [4]. Indeed, methyl groups provided by SAM are also used for methylating DNA sequences. It is well established that methylation of DNA at the carbon 5 of cytosines in the CpG islands of the promoter region of genes regulates their expression according to the percentage of promoter methylation. An increase in promoter methylation is associated with a decrease in the gene expression [1,5] and consequently to downregulation of protein level. The impact of promoter methylation in the regulation of gene expression can be explained considering the interference due to the methyl groups which negatively affect interaction with proteins (e.g., transcription factors, RNA-polymerase) involved in promoter recognition which is necessary to start gene transcription [4]. DNA methylation is regulated by the activity of specific enzymes: DNA-methyltransferase 1 (DNMT1), DNA-methyltransferase 3A (DNMT 3A) and DNA-methyltransferase 3B (DNMT 3B). DNMT1 has the role of maintaining DNA methylation during cell replication and guarantees the inheritance of the methylome during DNA replication and the permanence of the specific characteristics of the cell, while DNMTs 3A and 3B can catalyze the de novo methylation which depends to the impact of environmental stimuli (e.g., food, stress, xenobiotics, etc.) [1,2,3]. To ensure normal activity of the one carbon pathway, folic acid, vitamin B2, B6 and B12 are required; these micronutrients must be included in the diet because they represent the substrates used by the enzymes involved in the one carbon cycle to produce the methyl groups that the enzymes DMNTs and HMTs use for the proper DNA and histone methylation [5].

Gut microbiota also play an important function in epigenome remodeling [1]. The metabolites produced by gut microbiota can actively modulate the epigenome of the host, thus affecting his/her health status. Richness is the total number of bacterial species in the gut microbiome, while diversity is the number of individual bacteria from each of the bacterial species present in an individual’s gut microbiome. This relevant because richness and diversity of gut microbiota are the two major determinants of the health status. Indeed, bacteria can metabolize all kinds of fibers contained in food, such as vegetables, fruits and whole grains, producing short-chain fatty acids (SCFAs) (e.g., butyrate, propionate and acetate) which actively modulate gene expression and the epigenome in the colonocytes [5]. In particular, the SCFAs mediate both anti-inflammatory and metabolic responses, thus leading to positive systemic effects on lipid and carbohydrate metabolism [6,7]. Butyrate is a SCFA that promotes anti-inflammatory responses by interacting with receptors in colonocytes (e.g., GPR109A), immune cells (e.g., GPR43, GPR41) and adipocytes (e.g., GPR41) [8]. It inhibits the release of proinflammatory cytokines (e.g., IL6, IL12) and promotes anti-inflammatory responses (i.e., IL10). In doing so butyrate contributes to the maintenance of cellular homeostasis in the gut. Studies from animal models and cell lines highlight that the anti-inflammatory properties of butyrate also depend on the downregulation of the transcription factor *NF-kB* which controls the gene expression of proinflammatory cytokines [9]. Butyrate also plays a central role in lipid and glucose metabolism. By interacting with GPR43 and GPR41 receptors, it increases glucagon-like peptide 1 and peptide YY, decreasing glucagon production in the pancreas and increasing glucose uptake in muscle and adipose tissues. Butyrate increases fatty acid oxidation in muscle and decreases lipolysis in white adipose tissue resulting in a change in the body composition. Finally, butyrate can be also transported to the liver where it is metabolized to produce ATP. To support these functions more effectively a diet rich in fiber is necessary to modulate a healthy response by gut microbiota across the life span.

Considering the interplay between nutrition, gut microbiota and the epigenome, the first aim of this review is to describe the connections between early life nutrition (e.g., breastfeeding), childbirth (e.g., mode of delivery) and mode of care with gut microbiota modulation. Second, the role of maternal lifestyle in the programming of the infant’s health will be described, highlighting the impact of early life modulation of the epigenome. Finally, this review aims to share this fundamental information with all the health care professionals involved in maternity care, in order to promote the delivery of the best practices around birth.

## 2. Epigenetic Programming during the First 1000 Days of Life

Epigenetics etymologically means “on top of” or “in addition to genetics” to highlight the fact that it modulates gene expression without any change in the nucleotide sequence. Epigenetics contributes to cell differentiation during the first 1000 days of life—a period that includes forty weeks of pregnancy plus the first two years of life. DNA methylation switches genes off in order to differentiate more than 250 cell types that characterize tissues and organs in our body. Cells acquire an epigenetic memory of the neonatal exposome which is maintained throughout life.

Environmental cues (e.g., food, xenobiotics, stress, etc.) can regulate phenotypic diversity in the same genome and can influence the inheritance of epigenetic marks [10,11,12,13,14]. During this window of epigenetic plasticity, fetal growth, organ maturation and susceptibility to disease are all programmed.

Therefore, if the environmental cues are unhealthy (e.g., poor methyl group donor diet, endocrine disruption, stress, etc.), unwanted changes in the epigenome can occur, and alterations of gene expression could be observed later in life (e.g., at adolescent or adult age) increasing the risk for multifactorial environmentally driven diseases [4,5].

In support of this theory, several studies have found that maternally induced epigenetic programming and malnutrition (e.g., low Vitamin B12, low protein intake, high fat diet, exposure to endocrine disruptors, etc.,) during pregnancy can modulate body mass index, glucose tolerance, cardiovascular disease risk in the infant. The extent to which epigenetic programming is affected is influenced by both gender and the period of exposure to environmental factors. Severe maternal famine (about 400–800 kcal/die) exposure during the first trimester of pregnancy has been associated with changes in the promoter methylation of several genes (e.g., *IL10*, *INS-IGF2*, *GNASAS*, *LEP*, *ABCA1* and *MEG3*) [15,16,17]. Long-term effects include an increased risk of developing obesity in adult men [17] and a higher incidence of glucose intolerance in adult women [15,16,17]. Maternal adiposity and changes in the newborn DNA methylation have been observed in 19 cohorts within the PAGE consortium [18]. The consortium data found a correlation between promoter methylation at retinoid X receptor-α (*RXRA*) in umbilical cord blood and an increase of fat mass in 6- and 9-year-old children [19]. Correlations between maternal blood, cord blood metabolites (e.g., homocysteine, TMAO, 5meTHF, Vitamin B12, choline, methionine, SAH, etc.) and DNA methylation have been observed highlighting the key role of maternal metabolites in the modulation of the offspring’s epigenome [20]. Stress in utero and during early postnatal life, as well as childhood maltreatment, influence the hypothalamic–pituitary–adrenal axis resulting in fetal exposure to excess glucocorticoids [21]. Perkeybile and colleagues [22] demonstrated that a low level of early care leads to de novo DNA methylation at regulatory sites in the oxytocin receptor gene in the brain and blood and to the downregulation of the oxytocin receptor’s expression in the brain of infants [23]. This evidence demonstrates that both diet and maternal care are directly involved in the programming of adult health and act via epigenetics. Indeed, studies on animals show that stress due to maternal separation has been associated with modifications in fear regulation (e.g., an increased risk of developing anxiety, a higher consumption of palatable food). Remarkably, male rat offspring were more vulnerable than females to the effects of neonatal stress on anxiety-like behavior, as well as on food consumption [24,25]. Stress due to early life exposure to food pesticides (even at low dosage) in animals can modify neuronal development, leading to progressive neurodegeneration in adulthood. Aberrant epigenetic marks and biomarkers of neuronal damage can be inherited in offspring when both parents and only the mother are exposed to a low dosage of pesticide (near to the DL_50_) during their brain development [10,26].

Paternally induced epigenetic programming has been observed [27]. Offspring from prenatally undernourished fathers (where the mother was appropriately nourished), were more obese than offspring where both parents were eating a normocaloric diet before conception [28]. Studies on offspring born from fathers with a high-fat-diet (HFD) show changes in pancreatic DNA methylation [29]. Paternal low protein intake leads to altered expression of enzymes involved in the cholesterol metabolism in the liver in the offspring [30]. Pre-diabetes in fathers increases the risk of diabetes in their offspring [31]. Paternal exposure to nicotine or cocaine prior to conception influences the offspring’s drug resistance, affecting the expression of genes involved in the xenobiotic metabolism [32].

In summary, both epigenetic marks from mothers before and during pregnancy as well as those acquired from fathers can be inherited by the fetus. Malnutrition, alcohol, stress, smoking, xenobiotics and drugs can modify the epigenome of the future parents and the altered epigenetic marks potentially can be inherited by their offspring leading to epigenetic changes associated with the features of metabolic syndrome [27,33,34].

Moreover, further evidence derived from studies carried out on rodents demonstrates that small non-coding RNAs (sncRNAs) levels in sperm are influenced by the father’s life-style and environment. These sncRNAs contribute to the regulation of gene expression and endogenous mRNA transcription, and they play an important role in the post-fertilization zygote influencing early embryonic development [33]. Early postnatal stress has been associated with changes in sncRNAs profile in the sperm, and paternal high-fat diet can modify metabolic responses in the offspring also through spermatic sncRNAs [35].

Both intergenerational (i.e., subject directly exposed to the environmental stimuli) and transgenerational (i.e., subject was not directly exposed to the environmental insult) inheritance have been demonstrated in animal models [36]. Cardiac dysfunction induced by parental HFD persist for two subsequent generations in *Drosophila*, also when the offspring received a normal fat diet (NFD) [36]. A significant increase in whole-body H3K27me3 levels that persisted in the next NFD-fed generation has been observed, and the pharmacological inhibition of the H3K27 methyltransferase was able to block the intergenerational inheritance [37]. This study underlines another key aspect of epigenetics: its potential reversibility [38,39]. The plasticity of DNA methylation under different environmental conditions has been extensively evaluated in cell cultures, plants and animal models [38,39]. Human embryonic stem cells (hESCs) adapted to different culture conditions can have up- and downregulated genes that can reverse differently their DNA methylation profile [38].

The epigenetic impact of environmental cues and the epigenetic reversibility have their main plasticity during the first 1000 days of life: this is the “window of plasticity”, i.e., the period of life when the control of the future generations’ health can be properly addressed.

## 3. Gut Microbiota at Birth

It is yet to be established if any interaction with different bacteria via the placenta, umbilical cord or amniotic fluid actually occurs [40,41,42] and if it did what the consequences of such an interaction would mean for the health of the newborn. However, it has been established that is widely the composition of gut microbiota can be influenced by several factors such as genetics, ethnicity, food intake, environment, sex differences, gastrointestinal pH and oxygen/redox state [43,44]. Gestational age, mode of birth and breastfeeding can have an additional effect on gut microbiota composition in the first four years of life [45,46]. Preterm babies have been shown to have decreased diversity and lower concentration of *Bacteroides* and *Bifidobacterium* compared to infants born full term [47]. Their microbiota can be further reduced by antibiotic treatment and the environment of the intensive care unit [48]. Moreover, babies born by cesarean section (CS) also show a similar reduction in the diversity in their microbiota when compared to infants born vaginally [49].

Babies born by CS have more contact with the environmental microbiota (i.e., healthcare professionals) than with their mother’s microbiota (where exposure takes place in the vagina), resulting in a strong presence of epidermal *Staphylococci.* Dominguez-Bello et al., [50] identified that maternal vaginal bacteria (e.g., *Lactobacillus*, *Prevotella* or *Sneathia* spp.) were commonly found in infants born vaginally while maternal skin microbes (e.g., *Staphylococcus*, *Corynebacterium* and *Propionibacterium* spp.) were prevalent in infants born by CS. Shao et al., [51] observed in 596 full-term babies that birth by CS is associated with maternal transmission of *Bacteroides* strains and opportunistic pathogens (e.g., *Enterococcus*, *Enterobacter*, *Klebsiella* species), and that a similar picture can be observed in babies born by vaginally from mothers treated with antibiotics as well as in infants who are formula fed. Microbial composition in babies born vaginally consisted of up to 68.3% of *Bifidobacterium*, *Escherichia*, *Bacteroides* and *Parabacteroides* species [51]. Wampach et al., [52] similarly reported the profusion of *Bacteroides* and *Parabacteroides* in infants born vaginally, while an increase in the *Staphylococcus* was described following CS. Penders et al., [53] observed that in full-term infants born by CS, there is a 100-fold lower number of *Bifidobacteria* and *Bacteroides fragilis* when compared to those born vaginally. A 100-fold higher level of *Clostridium difficile* strains and increased level of *Escherichia coli* have been identified in the gut of babies delivered by CS than in those who were born vaginally and received exclusively breastfeeding [53]. A systematic analysis from Rutayisire et al., [54] highlights that CS is associated with a lower abundance and diversity of *Actinobacteria* and *Bacteroidetes* phyla and a higher abundance and diversity of the phylum *Firmicute* than in vaginally delivered infants. In addition, colonization of *Bifidobacterium* and *Bacteroides* genera, are present more frequently in vaginally derived infants when compared to babies delivered by CS, who in turn are preferentially colonized by *Clostridium* [54]. Moreover, investigation on gut microbiota by Liu et al., [55] on Chinese infants shows that vaginally delivered newborns harbor more *Acinetobacter* spp., *Bifidobacterium* spp. and *Staphylococcus* spp., while infants born by CS predominantly have *Citrobacter spp*., *E. coli* and *Clostridium difficile* strains. Shi et al. [56] observed additional differences in a Chinese cohort of infants, with an abundance in the *Propionibacterium* species in vaginally delivered babies, while in infants delivered by CS, there was a high presence of *Bacillus licheniformis*.

Gut microbiota are healthier and more diverse in babies born vaginally when compared with infants born by CS. In addition, babies born vaginally also demonstrate an increased richness and diversity in microbiota which are associated with a healthy phenotype. The presence of different strains of bacteria in the gut can be modulated by food intake and postnatal factors such as maternal diet, breast feeding, formula-feeding and weaning [43]. Breastfeeding increases the number of *Bifidobacterium* in the infant’s gut when compared to those who are formula-fed [43]. The crosstalk between host cell and gut microbiota has been widely demonstrated; Teng et al., [57] showed that ginger exosomes-like nanoparticles can modulate gut microbiota composition due to RNAs contained in the plant. In mice, an improvement of intestinal barrier function has been observed following the administration of ginger. The ginger exosomes-like nanoparticles contain miRNA that are used by gut *Lactobacillaceae*, inducing the production of the protective *IL-22* and enhancing gut barrier. Animal food (e.g., meat, egg, cheese) has been associated with a decrease of *Firmicutes* (*Roseburia*, *Eubacterium rectale*, *Ruminococcus bromii*) and an increase of *Enterobacteriaceae* (*Shigella* and *Escherichia*), while a high fiber diet promotes an increase in *Bacteroidetes* (e.g., *Prevotella*, *Xylanibacter*) and depletion of *Firmicutes* [58]. Polyphenols contained in fruit and vegetables promote the growth of beneficial pool of *Bifidobacterium*, *Lactobacillus, Akkermansia* and *Faecalibacterium* sp. and decrease in pathogenic organisms (such as *Helicobacter pylori*, *Staphylococcus* sp.) [59]. Vitamin C impact positively the gut microbiota microenvironment, increasing *Lactobacillus* and *Bifidobacterium* and decreasing *E. coli* [60]. Pre- and postnatal Vitamin D3 exposure modulates the profusion of bacterial taxa in the infant microbial gut population [61]. Vitamin D supplementation has been associated with beneficial bacterial genera (e.g., *Subdoligranulum*, *Ruminiclostridium*, *Intestinimonas, Pseudoflavonifractor*, *Paenibacillus*, *Marvinbryantia*) which have been associated with an antihypertensive function in later life [62]. Vitamins A and E show a positive influence on beneficial microbes, such as *Bifidobacteria, Akkermansia* and *Lactobacilli*) [59]. This evidence supports the existence of a direct link between diet and microbiome composition, which, as previously described, is a strong determinant of health.

The composition of gut microbiota can influence neuronal development and homeostasis in the infants [63,64]. The effect of stress on the gut–brain axis has been associated with changes in gut microbiota, alterations in brain derived neurotropic factor, behavioral changes, and it can even lead to anxiety and depression [63]. This evidence highlights that the composition of microbiota could represent a new strategy for the prevention of mental illness [65]. Studies on stressed adolescent rats treated with a preventive diet containing ω-3 polyunsaturated fatty acids (e.g., eicosapentaenoic acid, docosahexaenoic acid, docosapentaenoic acid) and vitamin A, did not show any decline in brain-derived neurotrophic factor expression in the hippocampus, and not even any change in the composition of microbiota, that was previously detected in the stressed rats [66].

Administering antibiotics has the potential to selectively alter the composition of gut microbes. The effect on both mother’s and child’s health of administering antibiotics during pregnancy or around the time of birth has not been completely elucidated. Several studies have been carried out to investigate the potential adverse effects of using antibiotics prenatally, during pregnancy or neonatally on the baby’s gut microbiome and the development of the infant immune system—possibly leading to childhood atopy, asthma, allergy and obesity [67,68].

In contrast, studies in which mice had their microflora remodeled by broad-spectrum antibiotics show that this treatment induces changes in regulatory T-cells conferring protection against experimental autoimmune encephalomyelitis (EAE), in animal model of CNS demyelinating disorders [69]. This tolerance was associated with reduced circulating proinflammatory cytokines and increased levels of IL-10 and IL-13, which are potent anti-inflammatory cytokines [70]. Antibiotic treatment also proved to be beneficial to children with regressive onset autism in the short term [71].Treatment for hepatic encephalopathy with rifaximin showed improved cognitive abilities posttreatment together with reduced endotoxemia and improved white matter integrity in patients [72].

Current research regarding the effect of antibiotic administration during pregnancy and birth gives insufficient attention to the confounding variables (such as type of antibiotic, dosing, mode of administration, etc.). In most studies, there is a lack of control for the timing of the use of antibiotics, the type and class of antibiotic and whether it is broad spectrum or not, the indication and the number of antibiotic courses administered [73,74].

In conclusion, gut microbiota composition at birth can be modulated by vaginal or CS birth, antibiotic administration and maternal diet. Since the presence of different strains of bacteria in the gut are associated with the production of metabolites able to positively modulate immune and metabolic responses in the infants, particular attention should be given by clinicians and healthcare professionals to promote gut microbiota diversity, maternal diet and mode of birth in order to maximize outcomes for mother and infant.

## 4. Mode of Birth

Significant progress in terms of surgical skills, blood transfusion, the development of antibiotics, etc., has reduced the mortality associated with CS and has reduced the risk to both mothers and neonates. As CS became a safer procedure, CS rates have increased worldwide (without commensurate improvements in perinatal mortality). For example, in Italy, CS rates in the 1980s were approximately 11% and had peaked to 38% by 2018. Similarly, in Australia, rates have increased from 11.8% to 47.4% across 81 hospitals with rates of 82.1% among 61,894 maternities with one previous CS [75]. This highlights the extent to which the first birth by CS of a repeat CS in subsequent pregnancies. The indications for CS have also changed over time as maternal request for CS in the absence of any medical indication (Cesarean Delivery on Maternal Request-CDMR) is becoming a clinically important factor. The extent to which maternal request for CS was influencing CS rates internationally led the American College of Obstetricians and Gynecologists (ACOG) to disseminate specific recommendations for obstetricians: *”in the absence of other indications for early delivery, cesarean delivery on maternal request should not be performed before a gestational age of 39 weeks; and, given the high repeat cesarean delivery rate, patients should be informed that the risks of placenta previa, placenta accreta spectrum, and gravid hysterectomy increase with each subsequent cesarean delivery”* [76].

An inappropriate use of surgery in childbirth has led not only to an increase in CS births, but also to a corresponding fall in vaginal birth after cesarean (VBAC) rates without any improvement in maternal or neonatal mortality [77]. Moreover, concerns are growing as to the short and long term adverse effects of CS [78], both for elective and emergency indications [79]. With respect to the mother adverse effects of CS include complications of surgery and anesthesia, the risk of hemorrhage requiring a blood transfusion, trauma due to intraoperative surgical injury, infection, pelvic adhesions, risk of placenta accreta syndrome, uterine rupture, and peri-partum hysterectomy. Adverse effects on the neonate include transient tachypnoea of the newborn, reduced breastfeeding rates and the consequent long-term risk associated with same, an increased risk of still birth and preterm birth in subsequent pregnancies [80].

Performing a VBAC is difficult as it remains prohibited in some countries contrary to the evidence and guidance produced by the National Institute of Health (NIH) and the American College of Obstetricians and Gynecologists (ACOG) who continue to recommend a “Trial of Labor after Cesarean” (TOLAC) for women who had one previous CS [81]. The safety of offering a VBAC to women following one previous CS has been demonstrated in a pan-European trial [81,82]. However, in practice, the trend of increasing CS that is occurring worldwide is difficult to reverse. Therefore, having a VBAC after a first CS in low risk women is highly encouraged, due to the potential beneficial epigenetic effects for the infant born vaginally. In contrast, elective/pre-labor CS is associated with altered short-term immune responses such as reduced expression of inflammatory markers in the newborn infant [80,83,84,85,86,87,88,89,90,91]. Infants born by elective CS also face a greater risk of developing immune diseases such as asthma, allergies, type 1 diabetes and coeliac disease [80,83,84,85,86,87,88,89,90,91].

Mode of birth is not only an obstetric issue as giving birth by CS affects the epigenetic state of different neonatal systems. In the case of CS birth, many babies are born before or at 39 weeks gestation, most mothers receive intraoperative antibiotics which has been shown to affect microbiota colonization of the infant’s gut which have been linked with a higher risk of specific diseases such as asthma, coeliac disease, obesity [92]. This is concerning when considered in contrast to the gut microbiota variation that exists in infants born vaginally at full term who are also breast fed.

Mode of birth does affect the epigenetic state of hematopoietic stem cells [93]. This may have important implications for health and disease in later life [79], and in turn may have an impact on short- and long-term health outcomes such as neural and behavioral development.

Of note, it has been shown that infants who were born by CS may experience cognitive and motor development delay at nine months of age [81]. Another study suggests that these infants show an increased risk of approximately 20% of having a diagnosis of autism spectrum disorder (ASD) [94]. A link seems to exist between CS and an increased risk ASD and attention-deficit/hyperactivity disorder (ADHD) not observed in infants born vaginally. Thus, the importance of mode of birth as a key factor in terms microbial and brain development warrants further investigation if we are to maximize all opportunities to have a positive impact on health outcomes from birth and across the lifespan [95]. Although this study has been supported by a recent meta-analysis [96] further studies are needed as the association between mode of birth and impairment of neuronal development has not been detected when using sibling controls, suggesting that this association is likely confounded by genetic and/or environmental factors.

## 5. Breastfeeding Versus Formula Feeding

Human breast milk is an optimal food for infants, not only because of its constituents, but also because of its dynamic nature, which changes to meet infant requirements as he/she grows. The variety of nutrients and bioactive molecules contained in breastmilk contribute positively to healthy growth and neurodevelopment. Several studies have shown that maternal nutritional status has an impact on milk composition, which in turn affects infant development [97].

Epidemiological studies on the long-term effect of breastfeeding on health have shown that breastfeeding in the first six months of life positively impacts on the infant’s anti-inflammatory responses, body mass index and blood pressure [98,99,100]. Furthermore, a decrease in the plasma level of IL6 has been measured in mothers who exclusively breastfeed their babies for more than six months [101]. Data from longitudinal studies report that exclusive breastfeeding for 3–12 months decreases from 20% to 28% the level of C reactive protein (CRP) in adulthood (28–32-year-olds) [98].

Observational studies have shown that a high protein intake associated with formula feeding is connected to rapid weight gain and obesity later in life in [99]. A multicenter European study on 1138 healthy infants showed a decrease in weight (e.g., length for age, weight for age, BMI) in those who were breastfed during their first year of life when compared with infants who were formula-fed. A positive reduction in body weight was observed when cow’s milk-based infant formula was decreased from high (2.9 and 4.4 g protein/100 kcal, respectively) to low protein content (1.77 and 2.2 g protein/100 kcal) [102,103]. The composition of breast milk is highly dynamic, and it varies across time. Leptin, ghrelin, adiponectin, insulin and more than 275 metabolites have been detected in breast milk [92,93], and changes in its composition from the 1st to the 6th month postpartum responds to infant requirements, particularly for central nervous system development. Long chain omega-3 fatty acid docosahexanoic acid (DHA), lutein, carotenoids and tocopherol are components of breast milk that have a key role for brain and retina development [104]. In a longitudinal study on infant *rhesus macaques*, Liu et al. [105] demonstrated structural brain differences that were capable of influencing brain development between breastfed and formula-fed animals. The presence of carotenoids and Vitamin E in formula milk was not able to mimic the efficacy of breast milk.

Longitudinal studies on children have shown that breastfed babies have healthier dietary patterns later in life, compared to children who were formula-fed [106,107]. The intake of vegetables can be modulated by the mother during pregnancy and lactation, as the nutrients from these can pass to the fetus/infant through the placenta and breastmilk [106,107]. Breastfeeding can also impact pubertal maturation [108]. A prospective study in 1237 girls, showed a later onset of breast development in girls who were breastfed compared to formula fed ones [108].

Exclusive breastfeeding (EBF) plays an important role in establishing the neonatal gut microbiota, leading to short and long-term benefits across different populations [109]. Although previous studies on the differences in gut microbiota between EBF and non-EBF infants have provided conflicting results, a meta-analysis has shown that, in the first six months of life, gut bacterial diversity, relative abundance of Bacteroidetes and Firmicutes and predicted microbial pathways related to carbohydrate metabolism are consistently lower in EBF infants [110]. In addition, infants display higher relative abundance of pathways related to vitamin and lipid metabolism and detoxification. In addition, there is reduced diarrhea-related gut microbiota dysbiosis associated with longer duration of EBF [110].

While EBF has been found to be beneficial for the composition of the gut microbial colonization in term infants, there is limited evidence about the influence of type of feeding on the gut microbiome of preterm infants [111]. Studies show that preterm infants with a diet consisting of at least 70% maternal milk have an increased gut microbial diversity in early life, with the highest abundance of *Bacillales*, *Lactobacillales* and *Clostridiales* when compared to other infants in different feeding groups. In preterm infants there is a high risk of dysbiosis of the gut microbiome as prematurity of the gut microbiota together with the preterm breast milk composition and the environment of the neonatal intensive care unit, pose a significant challenge in neonatal nutritional care [111].

Although the benefits of breastfeeding are widely accepted, challenges to find optimal ways to support mothers to continue to BF following initiation remain. Begley at al. [112] found that the timing of breastfeeding support was particularly crucial in the immediate postnatal period. In a model of care where midwives are perceived to be inaccessible, i.e., busy with other clinical activities, then this can lead to a situation where women are reluctant to seek help and support with breastfeeding. This has been associated with a negative impact on the continuation of breastfeeding on discharge, in particular, in a setting where community support for breastfeeding is almost non-existent. Some data are emerging that a midwifery-led continuity model of care may have a positive effect on the duration of exclusive breastfeeding at least up to 16 weeks postpartum. The midwifery led model in this trial included providing continuity of breastfeeding information and support in pregnancy, during birth, in hospital and following discharge in the form of home visits [113]. McFadden at al. previously concluded that consistent breastfeeding support tailored to women’s needs seems to increase the duration of exclusive breastfeeding [114]. Therefore, it seems that when support for breastfeeding is built in to the model of care, women can be identified from groups with particularly low breastfeeding rates for additional targeted support in order to increase the numbers of infants in these groups who would benefit immensely from the benefits of receiving breast milk.

In summary, breastfeeding has a key role in the modulation of gut microbial composition and as a consequence has a positive impact on health in the long-term. The duration of breastfeeding, whether its exclusive or not and maternal nutritional status while breastfeeding can have a significant impact on the infant’s health. Midwifery care in particular when targeted towards women who require additional support may have benefits to mothers and babies across the lifespan (Table 1).

## 6. Best Practice, Culture and Education

Birth is the outcome of a complex evolutionary process in which an intricate neurohormonal system mediated by oxytocin [115] plays a crucial role.

To date, our understanding of the role of mode of birth (e.g., vaginal birth vs. CS) is limited in terms of knowing if vaginal birth or CS can actually result in negative physiological consequences for infants, either in the short or in the long-term or both. It has been established that the normal process of labor, which often takes many hours, exposes the infant to many mechanical, hormonal and oxidative stresses potentially influencing physiology and life-long health. The three main mechanisms proposed to explain why mode of delivery, spontaneous, induced and/or augmented vaginal delivery versus CS, may affect neonatal development are: (1) exposure to varying levels of physical stress and stress hormone surges during birth; (2) differences in microbial colonization of the infant intestinal tract between vaginal birth and CS; and (3) epigenetic modifications of gene expression [4,116]. However, it is yet to be established if epigenetics can be also be modulated by a range of routine practices such as laboring lying down, being confined to bed with reduced mobility and continuous electronic fetal monitoring, in low risk mothers should be informed about the positive and negative factors able to impact the infant’s epigenome.

Birth in hospital with a concurrent increase in the medicalization of the birth process has resulted in an overuse of intrapartum interventions and a global rise in CS as “mode of birth” [117]. Therefore, place of birth may be a factor that is associated with maternal wellbeing and birth outcomes [118]. Healthy women who have the opportunity to birth in settings/facilities that offer sufficient space to stand/move around, have access to birth companions and the support of a midwife on a one-to-one basis, are associated with improved outcomes and fewer interventions [119].

However, further research is required to establish if a link exists between maternal and infant epigenetic outcomes and the level of intervention/interference in the childbirth process [120,121], including mode of delivery [116]. Therefore, further investigation of the biologic/physiological processes in healthy childbearing women and fetus/newborn who have undergone unnecessary maternity care interventions is required [122] to offer new insights from an epigenetic perspective.

## 7. Conclusions

The study of nutri–epigenetic processes highlights important potential changes in the epigenome related to mode of birth, maternal lifestyle, method of infant feeding, etc. that can develop our understanding of how adult health is programmed during the first 1000 days of life. The parental epigenome can be modified across the lifespan and the epigenetic marks of events that occur in utero can be inherited by progenies. Therefore, clinicians and healthcare professionals, who are offering care to families during pregnancy and childbirth should take the opportunity to inform parents of the long-term effects of an unhealthy diet and the benefits of breastfeeding across the lifespan. The positive impact of breastfeeding on gut microbiota differentiation and control of proinflammatory biomarkers later in life for the child and mother should be emphasized in order that women can make an informed choice for their own health and that of their newborn. Therefore, the importance of epigenetic research as a way of understanding the intergenerational impact of birth practices on the programming of the infant’s epigenome should not be underestimated.

Table 2 summarizes the highlights of this review.

## Figures and Tables

**Table 1:** Table summarizing the main findings of the positive impact of breastfeeding.

Parameter	Breastfeeding	Reference
Nutrients	>richness in bioactive compounds	[85,93,94,95,96]
Duration of breastfeeding ^1^	>anti-inflammatory Response	[86,87,88,89]
Effects in the adulthood	<[CRP] <[IL6] healthier dietary patterns	[87,97,98,99]
Protein intake	Adequate intake: <body weight <BMI	[90,91,92]
Brain health	Structural differences promoting brain development	[96]
Gut health	>microbiome diversity ^2^ >microbiome abundance ^2^ <episodes of diarrhea ^2^	[100,101,102,103]
Midwifery promotion of breastfeeding	>maternal compliance in breastfeeding	[104,105]

^1^ > 6 months; ^2^ in both in term and preterm infants.

**Table 2:** Table summarizing the main findings of this review.

Highlights
The epigenetic impact of environmental cues and the epigenetic reversibility have their main plasticity during the first 1000 days of life: the “window of plasticity” Environmental factors can modify the epigenome of the future parents The altered epigenetic marks from the parents potentially can be inherited by their upcoming offspring Gut microbiota composition at birth can be modulated by vaginal delivery or cesarian section Breastfeeding has a key role in the modulation of gut microbial composition as well as in the long-term impact on health The diversity of gut microbiota can guarantee the production of metabolites able to positively modulate immune and metabolic responses in the infants Clinicians and healthcare professionals, who are in close contact with the parents during pregnancy and childbirth, should take the opportunity to inform parents on the long-term effects of unhealthy diets

## Abbreviations

HATs	histone acetyltransferases
DNMT1	DNA-methyltransferase 1
HMTs	histone methyltransferases
SCFAs	short-chain fatty acids
RXRA	retinoid X receptor-α
HFD	high-fat-diet
sncRNAs	non-coding RNAs
NFD	normal fat diet
hESCs	human embryonic stem cells
EAE	experimental autoimmune encephalomyelitis
CS	cesarean section
VBAC	vaginal birth after cesarean
ACOG	American College of Obstetricians and Gynecologists
TOLAC	trial of labor after cesarean
ERCS	elective repeated cesarean section
ASD	autism spectrum disorder
ADHD	attention-deficit/hyperactivity disorder
CRP	C reactive protein
DHA	acid docosahexanoic acid
EBF	exclusive breastfeeding
